# Emerging Functions of Protein Tyrosine Phosphatases in Plants

**DOI:** 10.3390/ijms252212050

**Published:** 2024-11-09

**Authors:** Jing Xin, Chuanling Li, Xiaoqian Liu, Xueke Shi, Yu Sun, Jian-Xiu Shang

**Affiliations:** 1 Key Laboratory of Molecular and Cellular Biology of Ministry of Education, Hebei Research Center of the Basic Discipline of Cell Biology, Hebei Key Laboratory of Molecular and Cellular Biology, College of Life Sciences, Hebei Normal University, Shijiazhuang 050024, China; xinjing_0223@163.com (J.X.); xiaoqianliu95@163.com (X.L.); 15175130775@163.com (X.S.); yusun05@mail.hebtu.edu.cn (Y.S.); 2 Key Laboratory of Tropical Fruit Biology, Ministry of Agriculture and Rural Affairs, South Subtropical Crops Research Institute, Chinese Academy of Tropical Agricultural Sciences, Zhanjiang 524091, China; chuanlingzz@163.com

**Keywords:** plant, protein tyrosine phosphatase, growth and development, stress

## Abstract

Reversible protein phosphorylation, known as the “switch” of the cell, is controlled by protein kinases (PKs) and protein phosphatases (PPs). Based on substrate specificity, PPs are classified into protein serine/threonine phosphatases and protein tyrosine phosphatases (PTPs). PTPs can dephosphorylate phosphotyrosine and phosphoserine/phosphothreonine. In plants, PTPs monitor plant physiology, growth, and development. This review summarizes an overview of the PTPs’ classification and describes how PTPs regulate various plant processes, including plant growth and development, plant hormone responses, and responses to abiotic and biotic stresses. Then, future research directions on the PTP family in plants are discussed. This summary will serve as a reference for researchers studying PTPs in plants.

## 1. Introduction

Protein phosphorylation is an important post-translational modification that affects various aspects of target proteins, such as subcellular localization, stability, activity, and interaction with other proteins. Protein kinases (PKs) phosphorylate proteins by transferring the γ-phosphate group from ATP to one or more specific amino acid residues of target proteins; in contrast, protein phosphatases (PPs) catalyze the dephosphorylation of substrate proteins. Traditionally, PKs have been viewed as signaling regulators, with PPs considered constitutive “housekeeping” enzymes [1]. However, an increasing number of studies suggest that PPs are specialized and tightly regulated catalytic factors that act as key regulators by dephosphorylating target proteins, thus acting as molecular switches in signaling pathways [2].

Based on substrate-phosphorylated residues, protein sequences, and catalytic mechanisms, PPs are classified into four groups: phosphoprotein phosphatases (PPPs), metal-dependent protein phosphatases (PPMs), aspartate-based protein phosphatases (ASPs) and tyrosine protein phosphatases (PTPs) [2,3]. Among these, PPPs and PPMs, known as serine/threonine phosphatases (STPs), require metal ions for their activity and target serine/threonine residues. ASPs use an aspartate-based mechanism to dephosphorylate serine/threonine and tyrosine residues. PTPs use an active cysteine residue for their activity and are further categorized into low molecular weight PTPs (LMWPTPs), classical PTPs (PTPs), and dual-specificity PTPs (DsPTPs). The PTPs specifically dephosphorylate tyrosine residues, while DsPTPs can dephosphorylate serine/threonine and tyrosine residues [2,3].

While about one-third of the PTPs in animals are tyrosine-specific PTPs, most PTPs in plants belong to the DsPTPs family. Both classical PTPs and DsPTP family members share a highly conserved CX5R motif within the catalytic core regions: (V/I) HCxAGxGR (S/T) G (where “x” represents any amino acid). The cysteine residue within this motif is critical for catalytic activity, serving as a nucleophilic that replaces the phosphate group from the substrate to form a phosphatase intermediate, thus facilitating the hydrolysis reaction. The PTPs have highly similar secondary and tertiary structures in the catalytic site, and the substrate specificity is determined by sequences other than the catalytic domain [4,5,6]. More and more studies have addressed the significant roles of PTPs in plants, while an overall summary is unavailable.

This review focuses on the functions of PTPs in plants, including their role in plant growth and development, their relationship with plant hormone responses, how PTPs regulate abiotic stress, how they are regulated by plants under abiotic stress, and their involvement in biotic stress. Through an in-depth exploration of these processes, we aim to inspire new thinking about the role of PTPs in plants.

## 2. PTP Family in the Model Plant *Arabidopsis thaliana*

**Figure 1:**
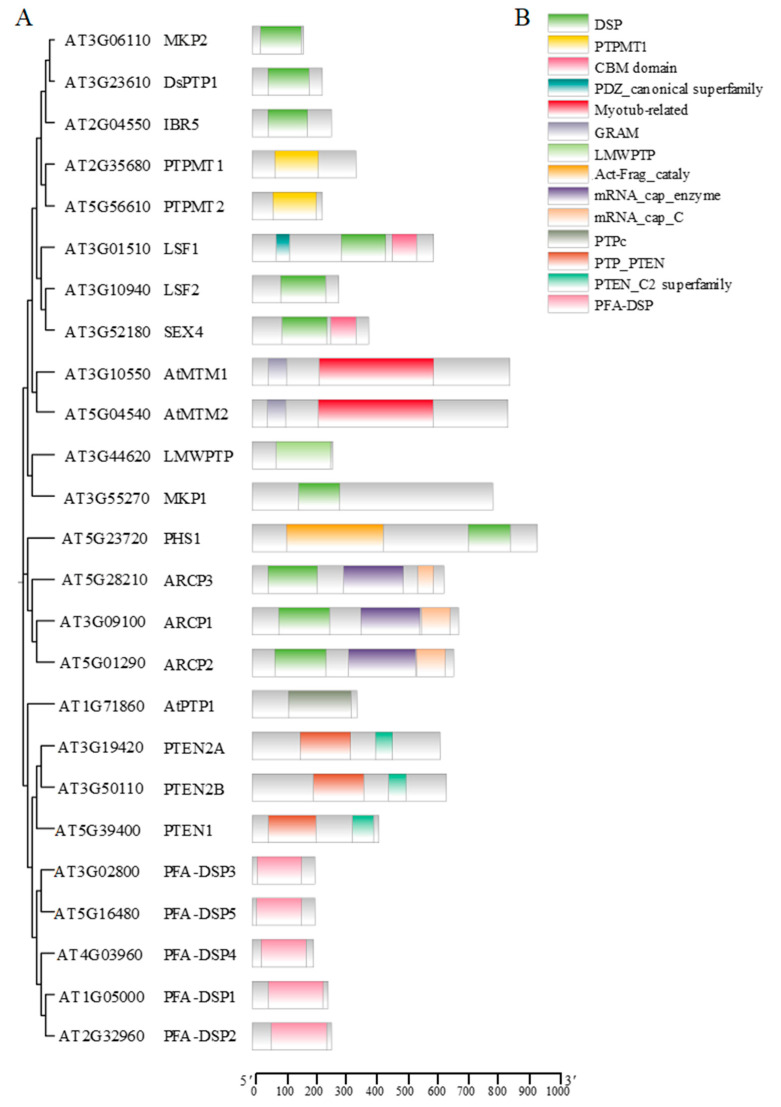
Phylogenetic tree and conserved motif of PTPs in *Arabidopsis*. (**A**) MEGA version 11.0.13 software generated the phylogenetic tree based on the 25 PTP family protein sequences. (**B**) PTP family with conserved motifs from NCBI were generated using TBtools-II version 2.119. Distinct conserved motifs are depicted using various colored boxes. The lower ruler indicates the amino acid sequence length from the 5′ end to the 3′ end of each protein.

The *Arabidopsis* genome encodes 25 PTPs, including 1 LMWPTP, 1 classical PTP named AtPTP1, and 23 DsPTPs [3] (Figure 1). The DsPTPs include five mitogen-activated protein kinase (MAPK) phosphatases (MKPs), five plant and fungi atypical dual-specificity protein tyrosine phosphatase (PFA-DSP 1–5), and other DsPTPs. These include the subfamily encoding phosphoglucan phosphatase genes (STARCH EXCESS4 [SEX4], LIKE SEX4 1 [LSF1] and LIKE SEX 4 2 [LSF2], the lipid phosphatase gene subfamily tumor suppressor phosphatase and tensin homologue deleted in chromosome 10 (PTEN) genes (PTEN1, PTEN2A, and PTEN2B), the myotubularin subfamily AtMTM (AtMTM1 and AtMTM2), the *Arabidopsis* mRNA capping enzyme family (ARCP1, ARCP2, and ARCP3), and protein tyrosine phosphatase localized to the mitochondrion (PTPMT1 and PTPMT2) [3].

MKPs include MKP1 (Mitogen-Activated Protein Kinases [MAPK] Phosphatase 1), MKP2 (MAPK Phosphatase 2), IBR5 (Indole-3-Butyric Acid Response 5), DsPTP1 (Dual-specificity Protein Tyrosine Phosphatase 1), and PHS1 (Propyzamide Hyper- Sensitive 1), all of which possess an AYLM-extended sequence following the characteristic PTP motif. These MKPs mainly dephosphorylate the tyrosine and serine/threonine residues of MAPKs, modulating various physiological processes in plants [7].

## 3. The Functions of PTPs in Plants

### 3.1. PTPs Regulate Plant Growth and Development

PTPs can dephosphorylate the phosphotyrosine and the phosphoserine/threonine residues and play essential roles in various physiological processes in plants.

The *mkp1* mutants in *Arabidopsis* are indistinguishable from that of the wild type at the early seedling stage. However, they show altered morphology approximately 3 weeks after germination, such as aberrant leaf development, early senescence, and dwarfism [8,9]. The loss-of-function mutant of *mkp1* in rice also exhibits a semi-dwarf phenotype [10], supporting the fact that MKP1 regulates plant height. Stomata on the plant epidermis control gas and water exchange. MKP1 is expressed preferentially in the stomatal cell lineage of the *Arabidopsis* epidermis. Loss of function of *MKP1* leads to clusters of undifferentiated cells instead of stomata, resulting in pavement cell patches [11]. MPK3 and MPK6 are two close members in clade A of the MAPK gene family. They are crucial regulators of stomatal development and patterning downstream of the MAPK kinase YODA [12]. Genetic analyses showed that MKP1 functions upstream of MPK3/6 but downstream of YODA. Biochemical experiments showed that MPK3/6 phosphorylation levels were increased in *mkp1* mutants. These studies suggest that MKP1 promotes stomatal cell fate transition by modulating MPK3/6 activation during the early stage of stomatal development [11,13]. In addition, MKP1 can function as a positive regulator of blue-light-mediated photomorphogenic development in *Arabidopsis*. Overexpression of *MKP1* enhances the blue-light-induced inhibition of hypocotyl elongation, results in more open cotyledons, increases pigment accumulation, and positively affects the expression of downstream blue-light-related genes. MKP1 interacts with and dephosphorylates MPK6, inhibiting its kinase activity and protein stability and thereby positively regulating blue-light-mediated seedling development in *Arabidopsis* [14].

Another member of MKPs, PHS1, is initially identified through a screen from *Arabidopsis* mutants displaying altered sensitivity to the microtubule-disrupting drug propyzamide. Under normal growth conditions, the dominant negative mutant *phs1-1* shows a significantly shorter root length than the wild type. It exhibits left-handed root twisting, accompanied by a less ordered and more fragmented cortical microtubule array [15]. PHS1 specifically interacts with MPK18 (MAPK18) from the 20 members of the *Arabidopsis* MAPK family in vivo. In *mpk18-1* loss of function mutants, cortical microtubules are moderately hyper-stabilized and can partially suppress the destabilized microtubule arrays phenotype in *phs1-1*. The PHS1–MPK18 signaling module is responsible for a phosphorylation/dephosphorylation switch that regulates cortical microtubule functions [16]. In addition, PHS1 plays a role during the floral transition by modulating the expression of the flowering activator *CO*, the floral integrator *FT*, and the repressor *FLC*. The loss function mutant *phs1-5* exhibits a late flowering phenotype under both long and short days, indicating that PHS1 positively regulates flowering time [17]. How PHS1 regulates the expression of these flowering genes and whether MPK18 works with PHS1 in this process requires further investigation.

The normal development of male and female gametophytes is crucial for sexual reproduction and the plant’s transition from generation to generation. AtPTEN, an active phosphatase that dephosphorylates phosphotyrosine and phosphatidylinositol substrates, is specifically expressed in pollen grains in *Arabidopsis*. When *AtPTEN1* is knocked down, pollen cells die after mitosis, resulting in plant sterility in plants [18]. Zhang et al. employed PTEN1 fusion with a YFP tag approach and discovered that PTEN1 regulates pollen tube growth through autophagy or vacuolar degradation by disrupting the dynamics of Phosphatidylinositol-3-phosphate [19]. Recently, our lab reported that mRNA capping enzymes ARCP1 and ARCP2 are critical for pollen development by affecting global gene expression levels, and heat stress triggers the degradation of mRNA capping enzymes and ultimately leads to male sterility [20].

In plants, carbon captured through photosynthesis is mainly stored as starch inside of the chloroplasts in the daytime. Various enzymes break down this starch at night or when photosynthetic processes are inactive. Then, the products are distributed to various plant organs and tissues to support growth and development. Typically, the starch molecule undergoes phosphorylation at the C3 and C6 sites. It is essential to eliminate these phosphate groups [21]. In addition to dual specificity phosphatase (DSP) domains, SEX4 and LSF1 possess a carbohydrate-binding module (CBM domain) and can bind to starch in vivo. Despite lacking a CBM, the homolog of SEX4, LSF2, can bind starch and is inside of starch granules [22,23,24] (Figure 1). Both SEX4 and LSF2 have phosphoglucan phosphatase activity. SEX4 regulates the removal of phosphate at the C6 position and the C3 position of starch, while LSF2 strongly prefers phosphate at the C3 position [22,23,24]. The *sex4* single mutant results in excess leaf starch, reduced plant growth, and delayed flowering, whereas the *lsf2* single mutant shows a slight increase in starch content compared to the wild type. The *sex4 lsf2* double mutants exhibit a more severe starch excess and growth inhibition phenotype than the *sex4* single mutant, suggesting that SEX4 and LSF2 are partially functionally redundant in the dephosphorylating of starch [22]. Ectopic overexpression of *OsSEX4* in *atsex4* rescues the starch accumulation phenotype in the mutant, indicating the conserved function of SEX4 in rice and *Arabidopsis*. *OsSEX4* deficiency significantly increases starch accumulation in leaves and straw. Using rice straw from *OsSEX4*-knockdown plants as a fermentation feedstock increases bioethanol yield [25]. Homologous *SEX4* genes have been identified in barley, cassava, and potato, which play a conserved role in regulating starch metabolism [26,27,28]. In contrast to SEX4 and LSF2, LSF1 is not an active glucan phosphatase, but it acts as a scaffold protein to promote starch degradation by binding β-amylase to starch granules [29,30].

**Figure 2:**
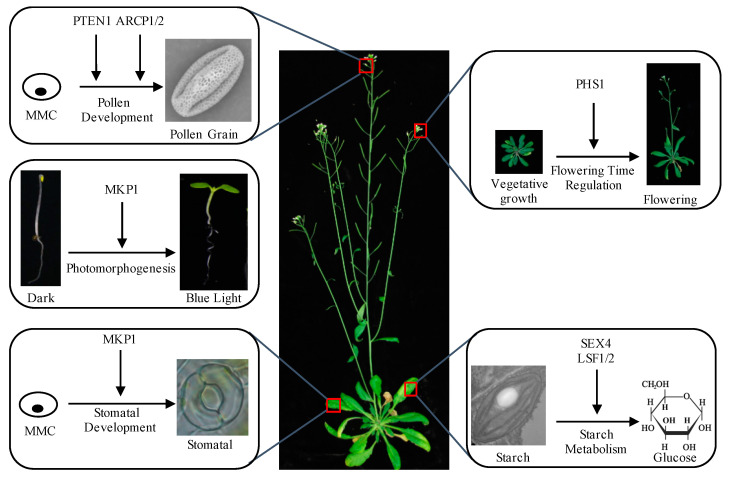
PTPs regulate plant growth and development. PTEN1 and ARCP1/2 are critical for pollen development. MKP1 plays a positive role in stomatal development and photomorphogenesis under blue light. PHS1 promotes flowering, while SEX4 and LSF1/2 are important for enhancing starch metabolism.

In summary, PTPs affect plant growth and development, including plant height, stomatal development, photomorphogenesis, flowering time regulation, pollen development, and starch metabolism. These processes are indispensable for maintaining plant growth and development (Figure 2).

### 3.2. PTPs and Plant Hormones

**Figure 3:**
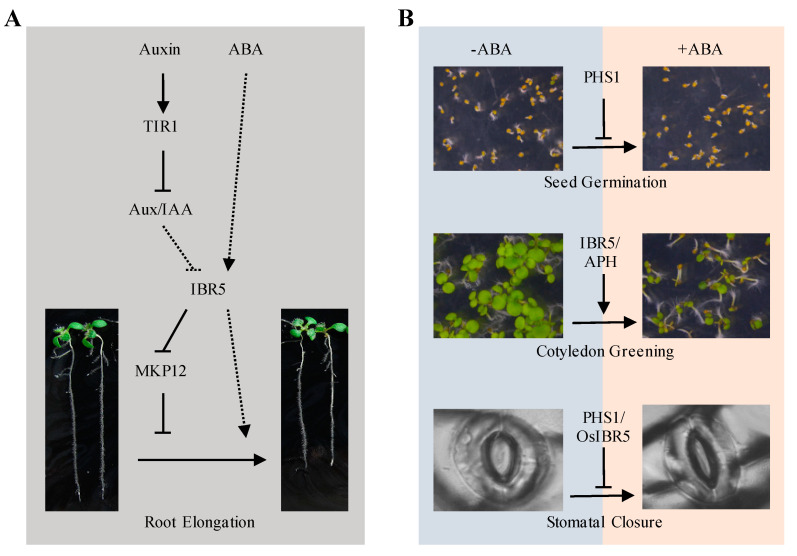
PTPs regulate ABA and Auxin responses. (**A**) IBR5 modulates ABA and auxin-mediated root elongation inhibition in *Arabidopsis*. (**B**) PTPs (PHS1, IBR5, APH in *Arabidopsis*, and OsIBR5 in rice) regulate ABA-inhibited seed germination, cotyledon greening, and stomatal closure. Solid arrows reflect direct positive influences, dashed arrows reflect indirect positive influences, solid T arrows denote direct negative effects, and dashed T arrows reflect indirect negative influences.

Plant hormones play important roles in plant growth and development, stress resistance, and completing the life cycle to generate offspring. Tyrosine phosphorylation sites have been identified through mass spectrometry under plant hormone treatment. Serine/threonine protein kinases and protein phosphatases of plant hormones’ signaling pathways can phosphorylate tyrosine residues or dephosphorylate tyrosine phosphorylation residues, respectively [5,31,32,33,34]. However, the involvement of specific PTPs in brassinosteroids, gibberellins, ethylene, and cytokinin is still unknown [35]. This review specifically focuses on the participation of PTPs in auxin and abscisic acid (ABA) responses, which have direct evidence (Figure 3).

#### 3.2.1. PTPs and Auxin Response

Auxin is one well-characterized plant hormone that acts as a growth regulator to control complex developmental processes throughout the plant’s life cycle [36].

It is reported that synthetic auxin caused a higher degree of tyrosine phosphorylation in *Arabidopsis* [37].

Research has identified IBR5 as a positive regulator in auxin response. The *ibr5* loss-of-function mutant showed reduced sensitivity to auxin-mediated inhibition of primary root elongation. *ibr5* mutant seedlings exhibited phenotypes similar to auxin-response mutants. They exhibited elongated primary roots, shortened hypocotyls, reduced lateral roots, increased leaf serration, and less auxin-response gene *DR5* accumulation [38]. The double mutant *ibr5 tir1*, obtained by crossing *ibr5* with the auxin receptor loss of function mutant *tir1*, exacerbated the reduced sensitivity phenotype to auxin in each parental mutant, indicating that the IBR5-mediated auxin response process is independent of auxin receptor TIR1. Examination of the inhibitor protein stability of Aux/IAA reporters in *ibr5* revealed that these proteins were not stabilized in *ibr5*, suggesting that IBR5 acts downstream of auxin recognition through the SCF^TIR1/AFB^-Aux/IAA complexes [39]. In addition, IBR5 protein phosphatase activity is required for full auxin response [39]. IBR5 specifically interacts with and dephosphorylates one MAPK, MPK12. The introduction of *MKP12* RNAi into *ibr5* mutants partially suppressed the reduced auxin sensitivity of *ibr5* mutants, highlighting the role of MPK12 as an IBR5 substrate in regulating the auxin response [40].

#### 3.2.2. PTPs and ABA Response

ABA regulates plant growth, development, and response to diverse environmental stresses [41].

Protein reversible phosphorylation plays a pivotal role in the ABA signaling pathway. In addition to serine/threonine phosphorylation, studies have emphasized the importance of tyrosine phosphorylation in this pathway. Knetsch et al. demonstrated that the ABA rapidly triggered activation of MAPK tyrosine phosphorylation and ABA-downstream target gene *RAB16* expression, which was completely inhibited by the exogenous application of a protein tyrosine phosphatase-specific inhibitor phenylarsine oxide (PAO), indicating positive regulatory functions of PTPs in ABA response [42]. Later, MacRobbie et al. discovered that PAO and another protein tyrosine phosphatase inhibitor, 3,4 dephosphatin (3,4-DP), impeded ABA-induced stomatal closure, further indicating that PTPs play positive regulatory roles in this process [43]. Subsequently, two-dimensional gel electrophoresis identified 19 proteins with altered tyrosine phosphorylation modifications after ABA treatment in *Arabidopsis* seeds, suggesting the involvement of reversible tyrosine phosphorylation in the ABA pathway [44].

Later studies demonstrated that PTPs play a role in ABA response. PHS1 functions as a negative regulator in ABA response. PHS1 loss of function mutant *phs1-3* exhibited increased ABA sensitivity. The *phs1-3* mutants showed a lower seed germination rate, enhanced ABA-induced stomatal closure, and higher regulation of ABA target genes compared to the wild type under ABA treatment [45]. When an ABA-induced *Fagus sylvatica* tyrosine phosphatase (FsPTP1) was overexpressed in seed dormancy accession Cape Verde Island ecotype, the transgenic seeds showed a reduction of seed dormancy and ABA insensitivity compared to the wild type, accompanied by a decrease in ABA-responsive genes’ expression. These results indicate that FsPTP1 is also a negative regulator of seed dormancy and ABA response [46].

Conversely, IBR5 positively regulates ABA response in *Arabidopsis*. The loss-of-function mutant of *ibr5* displays reduced sensitivity to ABA-mediated inhibition of primary root elongation and cotyledon greening [47]. However, overexpression of *OsIBR5* in tobacco disrupted ABA-induced stomatal closure, suggesting that IBR5 may play different roles in ABA responses in rice and *Arabidopsis* [48]. Recently, one study revealed a crucial function of LMWPTP, APH (AT3G44620), in regulating ABA-induced tyrosine phosphorylation in *Arabidopsis* [49]. APH functions as a protein tyrosine phosphatase. Dysfunction of APH resulted in less sensitivity to ABA in the post-germination growth and alteration of ABA-responsive genes compared to the wild type. APH represses the ABA-mediated protein tyrosine phosphorylation. Several splicing factors, posttranscriptional regulators, and a protein kinase (RAF9) are identified as the putative targets of APH [49].

In brief, pharmacological experiments have demonstrated that PTPs function as positive regulators in the ABA response; however, analyses of PTP mutants through physiological methods have found that some PTPs function as positive regulators in the ABA response and others function as negative regulators, illustrating the complex relationships between PTPs and ABA response. These studies indicate that PTPs are involved in ABA response in plants, but the molecular mechanisms of these regulations are unclear; whether PTPs dephosphorylate specific MAPKs or dephosphorylate some ABA signaling key components requires further investigation.

### 3.3. Regulating the Plant’s Response to Abiotic Stresses Through PTPs

Plants are sessile and are subjected to various abiotic stresses, including salinity, osmotic pressure, drought, ultraviolet radiation, oxidative damage, cold, and physical wounding, which harm plant growth and productivity. In addition to regulating plant growth and development, PTPs perform crucial functions in effectively combating multiple abiotic stressors (Table 1).

#### 3.3.1. PTPs Regulate the Salt Stress Response

Salt stress causes ionic stress and secondary oxidative stress. Plants employ various strategies to defend against salt stress, such as regulating ionic balance and mitigating cell damage caused by oxidative stress [75].

MKP1 modulates salt stress responses in *Arabidopsis* and wheat, although the specific responses observed in each case differ. The ectopic expression of the durum wheat MKP, TMKP1, enhances salt stress tolerance in yeast [76]. Similarly, transgenic *Arabidopsis* plants with heterologous overexpression of TMKP1 exhibit higher germination rates than wild-type plants under salt stress. The enhanced salt tolerance observed in the *TMKP1* transgenic seedlings is associated with elevated antioxidant enzyme activities, including superoxide dismutases, catalases, and peroxidases, which leads to a reduction in malondialdehyde (MDA), superoxide anion (O_2_^·−^), and hydrogen peroxide (H_2_O_2_) levels [55]. In contrast to the positive effects observed in wheat, MKP1 acts as a negative regulator of salt stress responses in *Arabidopsis*, as evidenced by the increased germination and seedling survival observed in *mkp1* loss-of-function mutants treated with salt compared to the wild type [55,56,76]. The precise molecular mechanism underlying this process remains an obstacle. The distinct function of MKP1 in salt stress in monocots and dicots may be caused by complex mechanisms controlling protein phosphatases in different plants.

Salt stress induces rapid depolymerization of microtubules (MTs) followed by the formation of a new MT network, which is better suited to survival under high salinity. The application of inhibitors of PTKs and PTPs affected MT depolymerization in salt stress [54]. MKP1 facilitates MT depolymerization under salt stress, thereby enhancing plant salt tolerance. Under salt stress, the expression of *MKP1* and the other four *MKP* genes (*MKP2*, *DsPTP1*, *IBR5*, and *PHS1*) is upregulated by a histone, H2B monoubiquitination (H2Bub1). Overexpression of *MKP1* effectively rescues the increased salt sensitivity of *h2bub1* mutants and alleviates the delayed microtubule depolymerization induced by salt treatment. In addition, the amelioration of the salt hypersensitivity in *h2bub1* by *MKP1* overexpression is mediated by the inactivation of MPK3 and MPK6 [54]. In brief, the PTP-MPK3/6 module is regulated by H2Bub1 to facilitate MT depolymerization under salt stress. However, the effect of MPK3/6 on cytoskeletal stability needs further study.

PHS1 is also involved in microtubule depolymerization under salt stress. Consisting of a MAPK phosphatase domain and a Mn^2+^-dependent atypical kinase domain, the atypical kinase domain of PHS1 is embedded in the MAPK phosphatase domain [57] (Figure 1). Under normal conditions, PHS1 interacts with and dephosphorylates MPK18 in the cytoplasm, as mentioned above, and the protein phosphatase activity of PHS1 suppresses its kinase activity, preventing the phosphorylation of α-tubulin. Salt stress can inhibit the phosphatase activity of PHS1, activated MPK18, and other unknown MAPKs may activate the tubulin kinase domain of PHS1. The active kinase form of PHS1 phosphorylates the conserved Threonine 349 of α-tubulin, leading to insufficient microtubule polymerization capability and compromising microtubule stability [16,57].

When mutated, another member of the MKP subfamily, *IBR5*, showed reduced sensitivity to salt stress and ABA-induced inhibition of cotyledon greening [47]. ABA is crucial in the response to salt stress, as important small molecules regulate metabolism. Further investigation is required to determine whether the role of IBR5 in the salt stress response depends on ABA.

Salt stress increases the transcription levels of tyrosine-specific protein phosphatases *AtPTP1* in *Arabidopsis* and *PdPTP1* in poplar [58,61]. Overexpression of *PdPTP1* leads to a significant accumulation of Na^+^, H_2_O_2_, and O_2_^·−^, accompanied by a decrease in K^+^ levels and activity of antioxidant enzymes, thus impairing the restoration of cellular ion and reactive oxygen species (ROS) homeostasis. These investigations suggest that PdPTP1 negatively modulates the plant’s response to salt stress. While PdPTP1 directly interacts with PdMPK3/6 in vitro and in vivo, further investigation is required to determine whether PdPTP1 dephosphorylates PdMPK3/6 to participate in salt stress in polar [61].

In addition to MKPs and the specific PTP, our laboratory found that the PFA-DSP subfamily, named AtPFA-DSP3/DSP5 (referred to as DSP3/DSP5), can also dephosphorylate MPK3/6 and negatively regulate the salt stress response in *Arabidopsis* [62,63]. Compared with the wild type, the *dsp3* and *dsp5* single mutants exhibited enhanced salt tolerance, with higher seed germination, chlorophyll content, survival rate, and lower ion leakage and ROS levels. In particular, the protein phosphatase activity of DSP3 was required for its function in the salt response. DSP3 interacted with and dephosphorylated MPK3 and MPK6. Genetic analysis of a *dsp3 mpk3* double mutant emphasized that the effect of DSP3 on salt stress depends on MPK3 [62,63].

In cotton, the *GhDsPTP3a*-silenced mutant exhibited a higher survival rate, longer primary root length, and less Na^+^ accumulation than the wild type after salt treatment. Ectopic expression of *GhDsPTP3a* in *Arabidopsis* reduces salt tolerance with less leaf greening, higher sodium Na^+^ accumulated, and lower primary root elongation under salt stress. These data indicate that GhDsPTP3a plays a negative regulatory effect on the salt stress response. Further studies revealed that GhDsPTP3a regulates plant salt stress by directly interacting with and dephosphorylating the salt-stress-induced phosphorylated annexin GhANN8b, thus modulating Ca^2+^ influx and Na^+^ efflux [64].

In summary, PTPs play a critical role in microtubule depolymerization, ion balance, and intercellular ROS homeostasis under salt stress through their interaction with MAPKs. In addition to classical substrate MAPKs, PTP dephosphorylates annexin to regulate ion homeostasis during salt stress.

#### 3.3.2. Regulation of Osmotic Stress Response by PTPs

Osmotic stress, which results from an imbalance in osmotic potential between plants and their environment, triggers many physiological changes at the cellular level, including changes in turgor, cell wall stiffness and integrity, membrane tension, and cell fluid volume, and plants may sense some of these stimuli and trigger downstream responses [77]. Proteins modified by kinases and protein phosphatases are essential in this process.

Osmotic stress suppresses the *IBR5* expression level in *Arabidopsis*. The *ibr5* mutant, which is less sensitive to osmotic stress, inhibited cotyledon greening in seedlings [47]. Osmotic stress induces the expression level of *DsPTP1* in seeds and seedlings in *Arabidopsis*. The *dsptp1* mutant exhibits increased seed germination rates and longer primary roots under osmotic stress compared to the wild type, and the complement lines can rescue the mutant phenotype of reduced sensitivity to osmotic stress. The *dsptp1* mutant shows increased proline accumulation, increased antioxidant enzyme activity, decreased MDA content, and reduced ion leakage rates under osmotic stress. Furthermore, *dsptp1* displays downregulated expression of the ABA synthesis gene *NCED3* and upregulated the ABA catabolism gene *CYP707A4*, reducing ABA accumulation in plants under osmotic stress [67]. The upregulation of ABA-positive regulators *ABI3* and *ABI5* was markedly reduced. The upregulation of ABA-negative regulator *ABI1* was higher in *dsptp1* mutant plants than in the wild-type plants following mannitol treatment. Consistent with this, *dsptp1* mutants showed reduced sensitivity to ABA-inhibited cotyledon greening and primary root elongation. These data suggest that DsPTP1 plays a role in osmotic stress by regulating ABA biosynthesis and signaling pathways. DsPTP1 interacts with and dephosphorylates MPK4 [65], but whether DsPTP1 is involved in osmotic stress regulation through dephosphorylating of MPK4 requires further investigation.

#### 3.3.3. Regulation of Drought Stress Response in Plants by PTPs

Drought is a major environmental stress that affects the growth and development of plants. Under drought stress, stomatal pores located on the plant epidermis regulate water loss through transpiration, and reversible protein phosphorylation participates in the regulation of stomatal aperture [78].

Studies suggest that PTPs are involved in the plant’s response to drought stress. Drought induces the expression of *OsIBR5*. Overexpression *of OsIBR5* in tobacco plants reduced sensitivity to ABA-induced stomatal closure, which resulted in a hypersensitive response of the transgenic tobacco to drought stress. OsIBR5 interacted with tobacco MAPKs (wound-induced protein kinase [WIPK] and salicylic acid [SA]-induced protein kinase [SIPK], homologs of AtMPK3 and AtMPK6, respectively) in yeast two-hybrid assays. Drought-induced WIPK activation was significantly reduced in *OsIBR5* overexpressing tobacco plants compared with the control, suggesting that OsIBR5 may negatively regulate MPKs and compromise the tolerance of transgenic tobacco to drought stress [48].

OsPFA-DSP1, which has PTP activity in vitro, is also induced by drought. When *OsPFA-DSP1* was ectopically expressed in tobacco or rice, the transgenic plants showed decreased sensitivity to ABA-induced stomatal closure and increased sensitivity to drought stress. This phenomenon suggests that OsPFA-DSP1 may regulate the ABA signaling pathway to defend against drought [68].

Plants ectopically expressing *GhDsPTP3a* exhibit increased sensitivity to drought stress, with more water loss than control plants. Meanwhile, the *GhDsPTP3a*-silenced plants display enhanced tolerance to dehydration and PEG-mimicked drought stress and have less water loss than control plants. The findings indicate that GhDsPTP3a plays a pivotal role in salt stress, as mentioned above, and it is also integral to the drought stress response in cotton [64].

The *Arabidopsis* genome encodes two MTM genes, AtMTM1 and AtMTM2, localized on the cis-Golgi and ER membranes [69]. MTMs are lipid phosphoinositide 3-phosphate phosphatases, and they use phosphatidylinositol 3,5-biphosphate as substrates to produce phosphatidylinositol 5-phosphate (PtdIns5P). Dehydration and ABA treatment increase the transcript level of *AtMTM1*, increasing the level of PtIns5P, suppressing ROS accumulation, and inhibiting stomatal closure. The loss function of *AtMTM1* showed less sensitivity to drought stress [79]. Conversely, AtMTM2 does not affect PtdIns5P levels under drought stress but promotes stomatal closure by facilitating ROS accumulation. These findings indicate that AtMTM1 and AtMTM2 act oppositely to regulate ABA-induced ROS accumulation, thereby maintaining intracellular homeostasis under drought stress [70,79].

#### 3.3.4. PTPs Regulate UV-B Stress Response in Plants

Light provides plants with energy for photosynthesis and is a key environmental signal regulating plant development. UV-B, a component of sunlight with a wavelength of 280–315 nm, is sensed at low doses by the plant photoreceptor UVR8, which triggers light signaling pathways to regulate plant growth, development, secondary metabolism, and adaptation to light. Conversely, exposure to high levels of UV-B induces UV-B stress, which inhibits plant growth and disrupts normal development [80].

MKP1 helps plants cope with UV-B stress [71]. The *mkp1* mutant showed increased sensitivity to UV-B stress and displayed hyperactivity of the UV-B-stress-activated MPK3 and MPK6 in *Arabidopsis*. Conversely, the *mpk3* and *mpk6* mutants showed increased tolerance to UV-B stress, and the *mpk3 mkp1* or *mpk6 mkp1* double mutant partially alleviated the UV-B stress hypersensitivity of the *mkp1* mutant. These results suggest that MKP1 mediates the dephosphorylation of MPK3 and MPK6 to protect against UV-B-induced cell death under UV-B stress. This MKP1-mediated UV-B stress pathway does not require UVR8, and MKP1 is unnecessary for UVR8-induced photomorphogenesis. Nevertheless, UVR8-dependent exposure to UV-B reduces MAPK activation upon UV-B stress. These data suggest that the UV-B-mediated photomorphogenic and stress-responsive pathways are independent. However, both MKP1 and UVR8 contribute to plant survival under simulated sunlight. These findings support the notion that UVR8-mediated acclimation promotes UV-B-induced defense measures, while MKP1-regulated stress signaling activates when UV-B protection and repair are insufficient and damage occurs. The coordinated activity of these two mechanisms is critical for plants’ UV-B tolerance [71]. Furthermore, MKP1 is a phosphoprotein, and UV-B exposure can cause the accumulation of MKP1 phosphorylation and protein stability, adding a layer of regulation to MAPK signaling in plants [72].

#### 3.3.5. Other Stress Responses Regulated by PTPs in Plants

In addition to their involvement in the abiotic stresses mentioned above, PTPs are also involved in genotoxic stress, oxidative stress, cold, and mechanical wounding. MPK3 and MPK6 are rapidly but transiently activated in plants exposed to genotoxic stress. Mutants of *MKP1* showed hypersensitivity to genotoxic and increased levels of MPK6 activation induced by genotoxic agents in *Arabidopsis* [56]. *MKP2*-suppressed plants were hypersensitive to ozone and showed prolonged activation of MPK3/6 in *Arabidopsis* [73]. The *ibr5* mutant seedlings showed hypersensitivity to oxidative stress induced by methyl viologen in *Arabidopsis*, while overexpression of *OsIBR5* in tobacco plants results in hypersensitivity to oxidative stress [47,48]. These results suggest that MKP1, MKP2, and IBR5 regulate the cellular response to genotoxic stress and oxidant challenges, respectively. The *ibr5-7* mutation suppresses the chilling-induced defense responses of *chs3-1* [74]. IBR5 interacts with the TIR domain of CHS3 and forms a complex with chaperone proteins HSP90 and SGT1b (suppressor of the G2 allele of *skp1*) to stabilize CHS3 protein. During wounding, such as cutting a leaf into pieces with a razor blade, the *OsMKP1* expression level is induced, which acts as a negative regulator by inactivating OsMPK3 and OsMPK6. Under normal growth conditions, the kinase activities of OsMPK3 and OsMPK6 are hyper-activated in *osmkp1* mutants compared to the wild type. Physical damage rapidly induces OsMPK3 and OsMPK6 activity in wild-type and *osmkp1* mutants, with a more pronounced effect observed in the *osmkp1* mutants [10]. In the orchid *Phalaenopsis amabilis*, the expression of the *PaPTP1* gene is mainly in floral organs and induced by mechanical wounding, indicating the potential role of PaPTP1 in the regulation of the development of the orchid flowers and response to wounding [81].

#### 3.3.6. How PTPs Are Regulated Under Abiotic Stress

PTPs regulate plant growth and development and respond to various stresses. Plants finely control PTPs, e.g., the transcriptional levels of *PTPs* are induced or suppressed under various stresses, as mentioned above, and plants also regulate the activity of PTPs through posttranscriptional regulations (Table 1).

Phosphorylation of MKP1 and MKP2 by their substrate MPK6 enhances their protein phosphatase activities in *Arabidopsis* [51,73]. This regulatory mechanism is also observed in other plants; co-incubation with substrates, such as SIPK or TaMPK3, significantly promotes the protein phosphatase activity of NtMKP1 in tobacco or TaMKP1 in wheat, respectively [52,82]. In addition, the protein phosphatase activities of MKP1 and DsPTP1 are influenced by calcium, calmodulin, and 14-3-3 proteins [53,66,83]. H_2_O_2_ reversibly inactivates AtPTP1 without affecting its protein stability [60]. Under nitric oxide (NO) treatment, the enzymatic activity of AtPTP1 decreases. Moreover, Cysteine 265 (tyrosine phosphatase activity site) is identified as the *S*-nitrosylation site in vitro. Pre-*S*-nitrosylation modification may protect against oxidative modifications of AtPTP1 by H_2_O_2_ [59].

**Figure 4:**
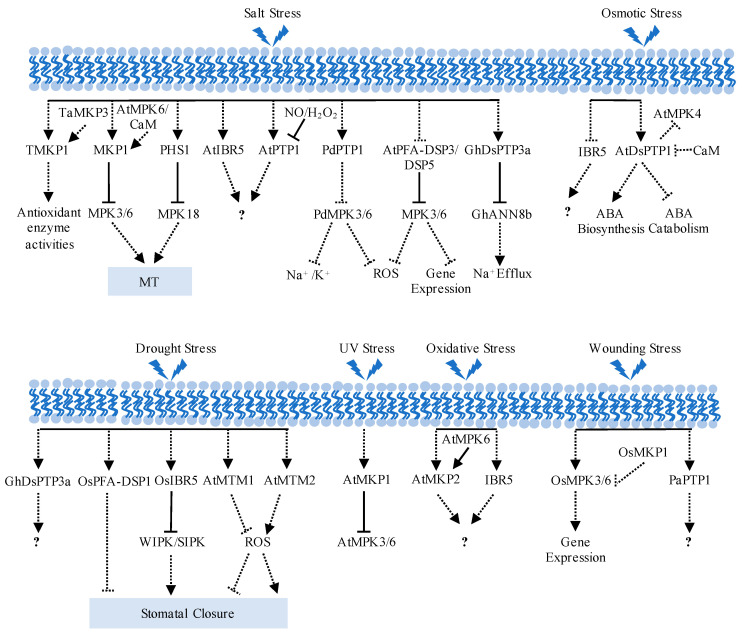
The relationship of PTPs and abiotic stresses. Under various abiotic stresses—such as salt, osmotic pressure, drought, UV radiation, oxidative stress, and wounding—PTPs help regulate microtubule rearrangement, stomatal closure, ROS homeostasis, ionic balance, ABA response, or gene expression to facilitate adaptation. Dashed arrows reflect indirect positive influences, solid T arrows denote direct negative effects, dashed T arrows reflect indirect negative influences, and “?” represents an unknown target.

In summary, PTPs are essential in responding to various abiotic stresses, such as salt, osmotic stress, drought, UV, oxidative stress, and wounding. Plants tightly regulate the activity of PTPs to defend against these harmful environmental factors (Figure 4).

### 3.4. Regulation of Plant Responses to Biotic Stress

Plants are exposed to many biotic stresses, including bacteria, fungi, viruses, parasitic worms, and insects, significantly impacting growth and yield. As sessile organisms, plants cannot escape these stresses but have developed strategies to protect themselves [84]. Several DsPTPs have been implicated in regulating pathogen-associated responses and resistance (Table 2).

#### 3.4.1. PTPs and Bacteria Pathogens

Plant diseases caused by bacterial pathogens significantly constrain crop production [91]. Plants possess a precise defense system that operates in a coordinated manner to mitigate or inhibit the infection process initiated by these pathogens [92].

*Arabidopsis* plants overexpressing *AtPFA-DSP4* reduce H_2_O_2_ accumulation and decrease photosynthesis in plants and then enhance plants’ susceptibility to *Pseudomonas syringae* pv. tomato DC3000 (*Pst* DC3000), indicating that AtPFA-DSP4 is a negative regulator in plant–pathogen response [85]. Like AtPFA-DSP4, the loss of function of *MKP2* plants exhibit enhanced resistance against a biotrophic pathogen *Ralstonia solanacearum* [86]. Similarly, MKP1 negatively regulates MPK6-mediated pathogen-associated molecular patterns response, thus modulating SA biosynthesis and resistance against bacteria. The *mkp1* (Col background) mutant enhanced expression of defense response markers, such as pathogenesis-related (PR) genes, and is resistant to virulent *Pst* DC3000 infection. Simultaneous deletion of *MKP1* and *PTP1* further inhibits the growth of *Arabidopsis*, repressing pathogen proliferation and intracellular spread and thereby enhancing plant resistance to *Pst* DC3000 [9,87]. Consistently, *Nicotiana tabacum* MAPK phosphatase1 (NtMKP1) can inhibit the biosynthesis of jasmonic acid or ethylene by inactivating two types of MAPKs, WIPK and SIPK, thus reducing plant resistance to the necrotrophic pathogen *Botrytis cinerea* [89]. These studies suggest that PTPs play a negative role against bacteria. However, *mkp1* mutants have reduced vascular resistance to *Xanthomonas oryzae* pv. *oryzaea* (*Xoo*) and *Xanthomonas campestris* pv. *campestris* (*Xcc*), indicating that MKP1 positively regulates vascular defense in plants. MKP1 negatively regulates the MPK3/6-mediated phosphorylation of the transcription factor MYB4, suppressing vascular lignification by inhibiting lignin biosynthesis and reducing vascular resistance to *Xoo* [88]. These studies shed light on tissue-specific defense responses of MKP1.

PTPs also play a crucial role in regulating resistance (R) proteins. The *ibr5* mutant partially suppresses autoimmune phenotypes and basal defense (*Pst* DC3000) response resulting from constitutive activation of R protein SNC1. IBR5 interacts with and promotes the accumulation of SNC1. IBR5 also controls disease resistance mediated by R proteins RPM1 and RPS4. The *ibr5* mutants are more susceptible to avirulent bacterial pathogens DC3000 (*avrRpm1*) and DC3000 (*avrRps4*) [74].

PTPs mainly coordinate the cross-interaction between plants and bacteria by influencing the intracellular ROS levels and SA biosynthesis and regulating the activity of MAPK or the stability of the R protein.

#### 3.4.2. PTPs and Fungal Pathogens

Fungal pathogens affecting plants constitute a highly varied and significant category of biological stressors that profoundly affect agricultural yield and financial results. Plants have developed an advanced immune receptor system to counter these fungal threats to identify and initiate defenses against these pathogens [93,94].

OsPFA-DSP2 plays a crucial negative regulatory role in the response to fungal infection. In transgenic rice with overexpressed *OsPFA*-*DSP2*, H_2_O_2_ accumulation was inhibited, and the expression of PR genes was suppressed, leading to the proliferation of the fungal pathogen *Magnaporthe grisea* [85]. In wheat, *TaMKP1* expression is rapidly induced upon fungal infection. TaMKP1 interacts with and dephosphorylates TaMPK3/4/6 to inhibit their kinase activity, negatively regulating wheat defense responses to wheat stripe rust caused by *Puccinia striiformis* f. sp. *tritici* (*Pst*) and powdery mildew fungi caused by *Blumeria graminis* f. sp. *tritici* (*Bgt*). Notably, the *Tamkp1* mutant exhibited increased resistance to fungal infestation, yet its agronomic traits exceeded those of the wild type. Generally, plant disease resistance and growth are antagonistic to one another. These findings indicate that TaMKP1 plays a role in maintaining a balance between plant disease resistance and growth, which could prove valuable for future agricultural applications [90]. These studies underscore the important role of PTPs in plant responses to various fungal infections.

**Figure 5:**
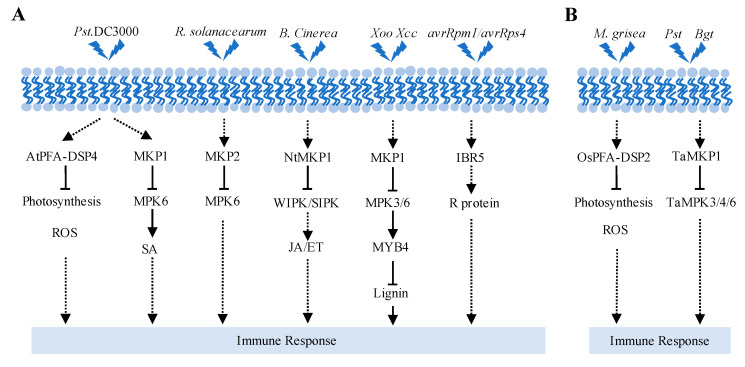
PTPs regulate various biotic stress responses. (**A**) PTPs regulate plant response to bacteria pathogens. (**B**) PTPs regulate plant response to fungal pathogens. *Pst* DC3000, *Pseudomonas syringae* pv. tomato DC3000; *R. solanacearum*, *Ralstonia solanacearum*; *B. cinerea, Botrytis cinerea*; *Xoo*, *Xanthomonas oryzae* pv. *Oryzaea*; *Xcc*, *Xanthomonas campestris* pv. *Campestris*; *avrRpm1* and *avrRps4* are avirulent bacterial pathogens of DC3000; *M. grisea*, *Magnaporthe grisea*; *Pst, Puccinia striiformis* f. sp. *tritici*; *Bgt*, *Blumeria graminis* f. sp. *tritici.* Solid arrows mean direct influences, dashed arrows reflect indirect positive influences, and solid T arrows denote direct negative effects.

The above studies suggest that PTPs participate in biotic stresses. Upon sensing external biotic stresses, plants mobilize PTPs, affecting physiological responses (photosynthesis) and intracellular ROS homeostasis, regulating MAPK cascades and plant hormones (such as SA, JA, and ET), or restructuring cell structure for defense against external biotic stresses (Figure 5). In addition to how MKP combats biotic stresses by regulating lignin synthesis, the specific molecular mechanisms of other PTPs involved in biotic processes remain to be further studied.

## 4. Conclusions and Prospects

PTPs play crucial physiological roles in plant growth and development, plant hormone responses, and responses to both abiotic and biotic stresses. Future research should focus on the following aspects: (1) Upon sensing alterations in the endogenous environment or external signals, how do plants quickly and efficiently regulate PTP activity to adapt to these changes and ensure survival? (2) Although it is widely accepted that the PTPs’ substrates are MAPKs, the potential involvement of other proteins as direct targets for PTPs necessitates further investigation and validation. (3) The role of PTPs under multiple distinct stress conditions is significant; elucidating how PTPs mediate different stresses simultaneously, such as the coexistence of drought and salt stress, will be an important avenue for future research. (4) The application of specific PTP mutations that enhance plant stress and disease resistance without negatively impacting normal growth represents another promising direction for agricultural advancements.

## Figures and Tables

**Table 1:** PTPs involved in various abiotic stresses.

Abiotic Stresses	Gene Names	Species	Substrates	Functions	Target of Regulation	Refs.
Salt stress	*MKP1*	*Triticum aestivum*	TaMPK3/TaMPK6	The germination rate and antioxidant enzyme activity in *TaMKP1* overexpression in plants increased compared to the wild type under salt.	TaMKP3 can enhance the protein phosphatase activity of TaMKP1.	[50,51,52]
		*Arabidopsis thaliana*	AtMPK3/6	The germination and seedling survival rates of the *mkp1* deletion mutant increased compared with the wild type under salt. MKP1 affects microtubule depolymerization during salt stress. Weaker depolymerization of cortical MTs induced by salt in *mkp1* mutant than the wild type.	Salt induces *MKP1* expression level. AtMPK6 can phosphorylate MKP1 and enhance its protein phosphatase activity. CaM can increase the phosphatase activity of AtMKP1.	[51,53,54,55,56]
	*PHS1*	*Arabidopsis thaliana*	AtMPK18	PHS1 mediates the depolymerization of microtubules under salt or osmotic stress by influencing the phosphorylation level of α-tubulin.	Salt increases the *PHS1* transcription level.	[16,54,57]
	*IBR5*	*Arabidopsis thaliana*	/	The *ibr5* mutant was less sensitive to salt stress and inhibited cotyledon greening in seedlings.	Salt induces *IBR5* transcription level.	[47]
	*PTP1*	*Arabidopsis thaliana*	/	/	Salt induces *PTP1* expression level. AtPTP1 protein activity is directly inhibited by H_2_O_2_ and nitric oxide exogenous treatments.	[58,59,60]
		*Populus deltoides*	PdMPK3/6	Na^+^, H_2_O_2_, and O_2_^·−^ levels were significantly accumulated, and the levels of K^+^ and activity of antioxidant enzymes were decreased in *PdPTP1* overexpression plants compared to wild type under salt.	Salt induces *PdPTP1* expression level.	[61]
	*PFA-DSPs*	*Arabidopsis thaliana*	AtMPK3/6	The *dsp3* and *dsp5* single mutant exhibited enhanced salt tolerance, higher seed germination, chlorophyll content, survival rate, and lower ion leakage and ROS levels than the wild type.	Salt induces DSP3 protein degradation.	[62,63]
	*GhDsPTP3a*	*Gossypium hirsutum*	GhANN8b	The *GhDsPTP3a*-silenced mutant exhibited a higher survival rate, primary root length, and less Na^+^ accumulation.	Salt triggers the expression of *GhDsPTP3a*.	[64]
Osmotic stress	*IBR5*	*Arabidopsis thaliana*	/	The *ibr5* mutant, less sensitive to osmotic stress, inhibited cotyledon greening in seedlings.	Osmotic stress suppresses *IBR5* expression level.	[47]
	*DsPTP1*	*Arabidopsis thaliana*	AtMPK4	The *dsptp1* mutant exhibits increased seed germination rates, longer primary roots, increased proline accumulation, decreased MDA content, reduced ion leakage rates, and downregulated expression of ABA synthesis gene *NCED3* and upregulated ABA catabolism gene *CYP707A4* than the wild type under osmotic stress.	*DsPTP1* is enhanced by osmotic stress. CaM inhibits the phosphatase activity of DsPTP1.	[54,65,66,67]
Drought stress	*IBR5*	*Oryza sativa*	SIPK/WIPK	Overexpression of *OsIBR5* in tobacco plants resulted in hypersensitivity to drought stress. The fresh weight of shoots and roots and the survival rate were lower. In contrast, the relative conductivity, stomata conductance, and leaf transpiration rate were higher in transgenic plants than in wild-type plants.	*OsIBR5* is induced by drought.	[48]
	*PFA-DSPs*	*Oryza sativa*	/	Ectopic overexpression of *OsPFA-DSP1* in tobacco transgenic plants showed increased sensitivity to drought stress.	Drought increases *OsPFA-DSP1* expression level.	[68]
	*GhDsPTP3a*	*Gossypium hirsutum*	GhANN8b	Ectopic-expressing *GhDsPTP3a* in *Arabidopsis* increased sensitivity to drought stress, with more water loss than the wild type.	Salt triggers the expression of *GhDsPTP3a*.	[64]
	*AtMTM1/* *AtMTM2*	*Arabidopsis thaliana*	/	AtMTM1 suppresses ROS accumulation and inhibits stomatal closure, while AtMTM2 promotes stomatal closure by facilitating ROS accumulation under drought.	Drought induces *AtMTM1* transcription levels.	[69,70]
UV stress	*MKP1*	*Arabidopsis thaliana*	AtMPK3/6	The *mkp1* mutant showed hypersensitivity to UV-B, with more leaf bleaching and dark pigmentation than the wild type. The MKP1-interacting proteins MPK3 and MPK6 are hyper-activated in *mkp1* through UV B stress.	UV-B promotes MKP1 phosphorylation and protein stability.	[71,72]
Oxidative stress	*MKP2*	*Arabidopsis thaliana*	AtMPK3/6	The *MKP2*-suppressed plants resulted in tissue collapse across the leaf blade under ozone treatment, and ozone-induced ion leakage is higher in the *mkp2* mutant than in the wild type.	AtMPK6 can phosphorylate MKP2 and enhance its protein phosphatase activity.	[73]
	*IBR5*	*Arabidopsis thaliana*	/	The *ibr5* mutant seedlings showed hypersensitivity to oxidative stress induced by methyl viologen.	/	[47]
		*Oryza sativa*	SIPK/WIPK	Overexpression of *OsIBR5* in tobacco plants results in hypersensitivity to oxidative stress. The relative chlorophyll content is lower in the *OsIBR5* transgenic plants than in wild-type plants.	*OsIBR5* is induced by oxidative stress.	[48]
Cold	*IBR5*	*Arabidopsis thaliana*	/	*ibr5-7* mutation suppresses the chilling-induced defense responses of *chs3-1*.	/	[74]
Wounding response	*MKP1*	*Oryza sativa*	OsMPK3/6	Wounding damage rapidly induces OsMPK3 and OsMPK6 activity in wild-type and os*mkp1* mutants, with a more pronounced induction observed in the *osmkp1* mutants.	Wounding induces *OsMKP1* expression level.	[10]

**Table 2:** PTPs involved in various biotic stresses.

Biotic Stress	Gene	Species	Substrates	Major Effects	Refs.
Bacteria	*PFA-DSP4*	*Arabidopsis thaliana*	/	Overexpressing *AtPFA-DSP4* plants reduce H_2_O_2_ accumulation and decrease photosynthesis compared to wild type under *Pseudomonas syringae* treatment.	[85]
	*MKP2*	*Arabidopsis thaliana*	MPK3/6	The loss function of *MKP2* plants exhibit enhanced resistance to *Ralstonia solanacearum* compared to the wild type.	[86]
	*MKP1*	*Arabidopsis thaliana*	MPK6	MKP1 negatively regulates MPK6-mediated pathogen-associated molecular patterns response, modulating SA biosynthesis and resistance against bacteria.	[87]
			MPK3/6	MKP1 negatively regulates the MPK3/6-mediated phosphorylation of the transcription factor MYB4, suppressing vascular lignification by inhibiting lignin biosynthesis and reducing vascular resistance to *Xanthomonas oryzae pv. oryzaea*.	[88]
		*Nicotiana tabacum*	WIPK/SIPK	NtMKP1 inhibits the biosynthesis of jasmonic acid or ethylene by inactivating two types of MAPKs, WIPK and SIPK, thus reducing plant resistance to *Botrytis cinerea*.	[89]
	*IBR5*	*Arabidopsis thaliana*	/	The *ibr5* mutants are more susceptible to avirulent bacterial pathogens DC3000 (*avrRpm1*) and DC3000 (*avrRps4*) than the wild type.	[74]
Fungal	*OsPFA-DSP2*	*Oryza sativa*	/	OsPFA-DSP2 overexpression plants inhibited H_2_O_2_ accumulation and the expression of PR genes, which led to the proliferation of the fungal pathogen *Magnaporthe grisea*.	[85]
	*TaMKP1*	*Triticum aestivum*	TaMPK3/4/6	TaMKP1 negatively regulates wheat defense responses to stripe rust and powdery mildew by dephosphorylating TaMPK3/4/6 to inhibit their kinase activity.	[90]
